# Correction: Research progress on nanomaterial-empowered electrochemical biosensors for the detection of cardiac troponin I

**DOI:** 10.1039/d5ra90093j

**Published:** 2025-08-08

**Authors:** Ning Zhang, Yuxin Zhu, Fachuang Li, Yusong Wang, Zheng Fu, Mengda Jia, Xiaoran Zhan, Wanqing Zhang

**Affiliations:** a Henan Institute of Technology School of Materials Science and Engineering Xinxiang 453003 China znzhangning121@hait.edu.cn; b School of Chemistry and Chemical Engineering, Henan Institute of Science and Technology Xinxiang 453003 China zhangwqzzu@163.com

## Abstract

Correction for ‘Research progress on nanomaterial-empowered electrochemical biosensors for the detection of cardiac troponin I’ by Ning Zhang *et al.*, *RSC Adv.*, 2025, **15**, 26473–26489, https://doi.org/10.1039/D5RA04555J.

The authors regret that the affiliations for Fachuang Li and Zheng Fu were incorrectly shown in the original manuscript; their affiliations should both have been *a* and not *b*. The corrected list of affiliations is as shown here.

The authors would also like to clarify that Zheng Fu was incorrectly labelled as a corresponding author in the original manuscript. Ning Zhang was also, incorrectly, not labelled as a corresponding author when they should have been. The corrected author list is as shown here.

Finally, the authors regret the email addresses for the corresponding authors were omitted from the original manuscript. These have now been included here.

The Royal Society of Chemistry apologises for these errors and any consequent inconvenience to authors and readers.

